# Discovery of Replicating Circular RNAs by RNA-Seq and Computational Algorithms

**DOI:** 10.1371/journal.ppat.1004553

**Published:** 2014-12-11

**Authors:** Zhixiang Zhang, Shuishui Qi, Nan Tang, Xinxin Zhang, Shanshan Chen, Pengfei Zhu, Lin Ma, Jinping Cheng, Yun Xu, Meiguang Lu, Hongqing Wang, Shou-Wei Ding, Shifang Li, Qingfa Wu

**Affiliations:** 1 School of Life Sciences, University of Science and Technology of China, Hefei, Anhui, China; 2 State Key Laboratory for Biology of Plant Diseases and Insect Pests, Institute of Plant Protection, Chinese Academy of Agricultural Sciences, Beijing, China; 3 Department of Computer Science and Technology, University of Science and Technology of China, Hefei, Anhui, China; 4 Department of Fruit Science, College of Agronomy and Biotechnology, China Agricultural University, Beijing, China; 5 Department of Plant Pathology and Microbiology, University of California, Riverside, Riverside, California, United States of America; 6 The CAS Key Laboratory of Innate Immunity and Chronic Disease, University of Science and Technology of China, Hefei, Anhui, China; The Ohio State University, United States of America

## Abstract

Replicating circular RNAs are independent plant pathogens known as viroids, or act to modulate the pathogenesis of plant and animal viruses as their satellite RNAs. The rate of discovery of these subviral pathogens was low over the past 40 years because the classical approaches are technical demanding and time-consuming. We previously described an approach for homology-independent discovery of replicating circular RNAs by analysing the total small RNA populations from samples of diseased tissues with a computational program known as progressive filtering of overlapping small RNAs (PFOR). However, PFOR written in PERL language is extremely slow and is unable to discover those subviral pathogens that do not trigger *in vivo* accumulation of extensively overlapping small RNAs. Moreover, PFOR is yet to identify a new viroid capable of initiating independent infection. Here we report the development of PFOR2 that adopted parallel programming in the C++ language and was 3 to 8 times faster than PFOR. A new computational program was further developed and incorporated into PFOR2 to allow the identification of circular RNAs by deep sequencing of long RNAs instead of small RNAs. PFOR2 analysis of the small RNA libraries from grapevine and apple plants led to the discovery of Grapevine latent viroid (GLVd) and Apple hammerhead viroid-like RNA (AHVd-like RNA), respectively. GLVd was proposed as a new species in the genus *Apscaviroid*, because it contained the typical structural elements found in this group of viroids and initiated independent infection in grapevine seedlings. AHVd-like RNA encoded a biologically active hammerhead ribozyme in both polarities, and was not specifically associated with any of the viruses found in apple plants. We propose that these computational algorithms have the potential to discover novel circular RNAs in plants, invertebrates and vertebrates regardless of whether they replicate and/or induce the in vivo accumulation of small RNAs.

## Author Summary

Viroids are a unique class of subviral pathogens found in plants, and they are difficult to identify since they are free circular non-coding RNAs and often replicate to low levels in host cells. We previously described the computational algorithm PFOR that discovers viroids by analyzing total small RNAs of the infected plants obtained by next-generation sequencing platforms. However, the algorithm written in PERL language is very slow, and viroid identification depends on the *in vivo* accumulation of extensively overlapping sets of small RNAs to target viroids. Here we report the development of PFOR2 that adopted parallel programming in the C++ language and was significantly faster than PFOR. We also describe a simple computational program that after incorporation into PFOR2 is capable of identifying viroids from deep sequencing of long RNAs instead of small RNAs. Moreover, we report the identification of Grapevine latent viroid (GLVd) and Apple hammerhead viroid-like RNA by the computational approach. Since our new algorithms do not depend on the analysis of viroid-derived small RNAs produced *in vivo*, it is possible to discover viroids in a wide range of host species including plants, invertebrates and vertebrates.

## Introduction

Viroids and a group of satellite RNAs (satRNAs) have single-stranded circular RNA genomes that range in size from 220 to 457 nucleotides (nt) [1]–[4]. These subviral pathogenic RNAs lack protein-coding capabilities and thus depend on either host-encoded DNA-dependent RNA polymerase (viroids) or helper virus-encoded RNA-dependent RNA polymerase (circular satRNAs) for replication [5], [6]. Viroids and circular satRNAs have been proven to be excellent biological models for studying non-coding RNAs (ncRNAs) and basic biology [3], [4]. The most notable examples include the discovery of RNA-directed DNA methylation (RdDM) in viroid-infected plants [7] and of the hammerhead ribozymes in viroids [8] and circular satRNAs [9] of plant viruses. Interestingly, recent studies have revealed the production of thousands of non-replicating circular RNAs (circRNAs) across species from Archaea to humans [10], [11]. These circRNAs are largely generated from back-spliced exons, in which splice junctions are formed by an acceptor splice site at the 5' end of an exon and a donor site at the downstream 3' end [10], [12], [13]. The functions of circRNAs are largely unknown, although a few circRNAs have recently been shown to play regulatory roles as, for example, microRNA sponges [10], [12], [13].

Viroids infect many crops and cause severe symptoms in susceptible hosts that result in economically important diseases [2]. However, the rate of discovery of the replicating circular RNAs is slow compared to the discovery of viruses [14]. For example, fewer than 40 viroid species, all of which infect higher plants, have been identified [15] since the first report in 1971 [1]. The slow rate of viroid discovery is often attributed to the technical difficulty involved in the purification and characterization of the naked non-coding circular RNAs that generally occur at low concentrations in the infected host [16]. We have recently described an approach for sequence homology-independent discovery of replicating circular RNAs by analyzing the total small RNA populations from samples of diseased tissues with a program known as progressive filtering of overlapping small RNAs (PFOR) [17]. The PFOR approach relies on the observations that rolling-circle replication of viroids and some satRNAs produces multimeric head-to-tail dsRNAs [5] and that continuous overlapping sets of small interfering RNAs (siRNAs) processed by Dicer [18]–[21] from the direct repeat dsRNAs accumulate to high levels in infected plant tissues [22], [23]. PFOR retains viroid-specific siRNAs for genome assembly by progressively eliminating non-overlapping small RNAs and those that overlap but cannot be assembled into a direct repeat RNA. Use of PFOR for the analysis of a grapevine small RNA library led to the discovery of a viroid-like circular RNA of 375 nt that encodes active hammerhead ribozymes in both plus and minus polarities [17]. However, it remains unknown whether the identified circular RNA can initiate independent infection.

Two major limitations of the first version of PFOR restrict its application in the discovery of circular RNAs. First, the iterative filtering of small RNAs that are not derived from a replicating circular RNA is a slow process and takes up more than 90% of the PFOR running time. Because PFOR was written in the explanatory PERL language, analyzing complex small RNA libraries using PFOR may take hours or days. Second, circular RNAs are not identifiable by PFOR if they neither replicate nor trigger Dicer-dependent siRNA production in a eukaryotic cell.

In this study, a considerably improved version of PFOR was developed by adopting parallel programming in the C++ language. The use of the new version of PFOR, designated PFOR2, led to the discovery of a new viroid from grapevine and a viroid-like RNA from apple tree. Moreover, a new program was developed and incorporated into PFOR2 for the discovery of distinct classes of circular RNAs, including those that neither replicate nor induce in vivo accumulation of Dicer-dependent siRNAs. We propose that the application of PFOR2 would speed up the discovery of novel circular RNAs and expand the list of known host species that can be independently infected by viroids.

## Results

### Development and performance of PFOR2

**Figure 1:**
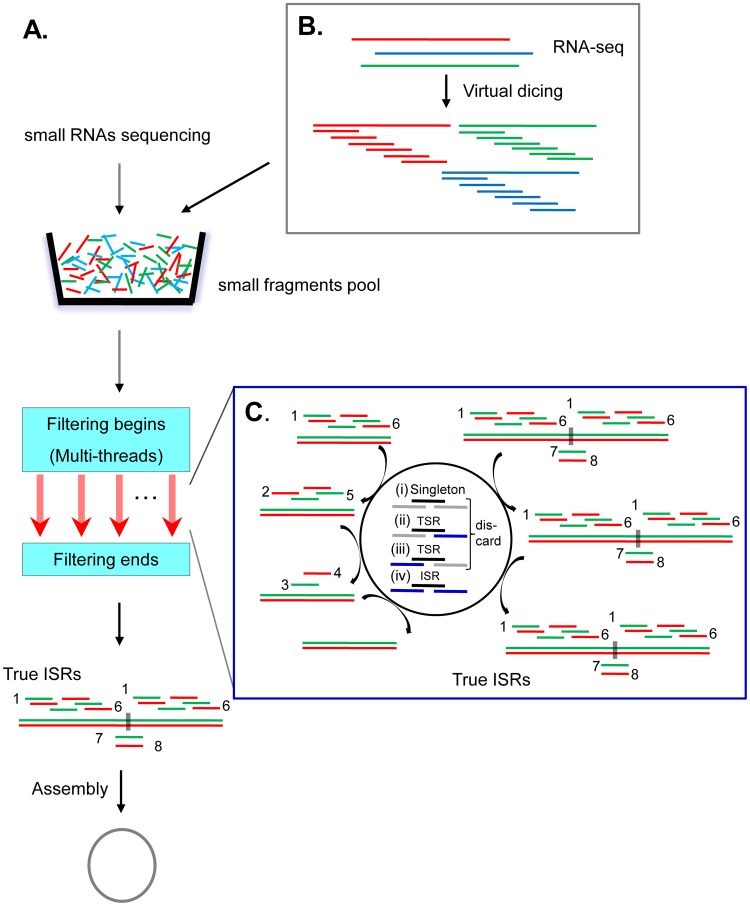
Illustration of the principles of the PFOR2 and SLS computational programs. (**A**) Flowchart showing the steps followed by PFOR2 for filtering all non-overlapping small RNAs (singleton) and terminal small RNAs (TSRs) with multi-thread processing and the assembly of the remaining true internal small RNAs (ISRs). (B) Schematic view of the virtual dicing of longer RNA reads into small overlapping sRNAs by SLS. (C) Close-up view of the filtering criterion of TSRs implemented in PFOR2. Each sRNA in the small fragments pool is placed into one of four groups based on the presence or absence of 5' and 3' overlapping sRNAs.

The computational algorithm of PFOR has been developed to identify replicating circular RNAs including viroids by deep sequencing of small RNAs [17]. A key step of PFOR algorithm is to separate terminal small RNAs (TSRs) from internal small RNAs (ISRs) in a small RNA pool. A small RNA is defined as an ISR if it overlaps at least one other small RNA at both ends larger than k-mer in the pool, whereas a TSR overlaps at least one other small RNA larger than k-mer in the pool at only one end of the TSR. The process of PFOR includes two main steps: filtering all non-overlapping small RNAs and terminal small RNAs (TSRs) with overlapping k-mers and assembling the remaining internal small RNAs (ISRs) predicted to derive from circular RNAs (Fig. 1C). Filtering TSRs derived from linear non-repeat precursor RNAs takes up more than 90% of PFOR running time. Therefore, to shorten the computing time required for filtering TSRs and to improve the performance of PFOR, PROR2 was developed by converting the previous algorithm written in the explanatory PERL language into the C++ language and adopting the parallel programming technology of OpenMP [24]. Because multiple shared memory filtering processes were performed concurrently in PFOR2, the TSR filtering process was expected to be faster than PFOR (Fig. 1A).

To test the performance of PFOR2, two publicly available small RNA libraries, in which both known viroids and viroid candidates have been verified by RT-PCR and Northern-blot hybridization, were used to compare the running time between PFOR and PFOR2. *Hop stunt viroid* (HpSVd), *Grapevine yellow speckle viroid* (GYSVd) and Grapevine hammerhead viroid-like RNA (GHVd RNA) were each identified, and their full-length genomic RNA sequences were obtained by both PFOR and PFOR2 from the grapevine tree sRNA library, which contains 4,701,135 reads of 18–28 nt in length (GEO accession no. GSM458928). However, PFOR2 required only 67 seconds and was 3.3 times faster than PFOR. The second sRNA library was from a peach tree infected with *Peach latent mosaic viroid* (PLMVd) and contained 7,862,905 reads of 18–28 nt in length (GEO accession no. GSM465746). Both PFOR and PFOR2 were again able to identify PLMVd and to recover the complete sequence. Instead of 2 hours by PFOR using a default k-mer of 17, PFOR2 required only 22 minutes. These results demonstrated that PFOR2 was indeed faster than PFOR in viroid discovery.

### Discovery of a novel viroid-like RNA from apple by PFOR2

PFOR2 was next applied to determine whether an apple plant with typical symptoms of apple scar skin disease contained new circular RNAs. The sRNA library was constructed from this apple plant, and 15,321,500 clean reads of 18–30 nt in length with a predominant size of 21 nt were obtained after deep sequencing (S1 Figure). Two putative circular RNAs were identified from the apple tree sRNA library by both PFOR and PFOR2, although PFOR2 was six times faster. The first RNA species was 333 nt and shared 96% sequence identity with a variant of *Apple scar skin viroid* (ASSVd) (accession no. KC110858) isolated previously from apple in China and was hence considered to be a new isolate of ASSVd. The second RNA species was 434 nt in length and showed no sequence similarity to any of the known entries in GenBank. Interestingly, the second predicted circular RNA also contained the conserved sequences found in hammerhead ribozymes as shown previously for GHVd RNA [17]. Thus, the second RNA identified from the apple sRNA library by PFOR and PFOR2 was tentatively designated as apple hammerhead viroid-like RNA (AHVd-like RNA).

**Figure 2:**
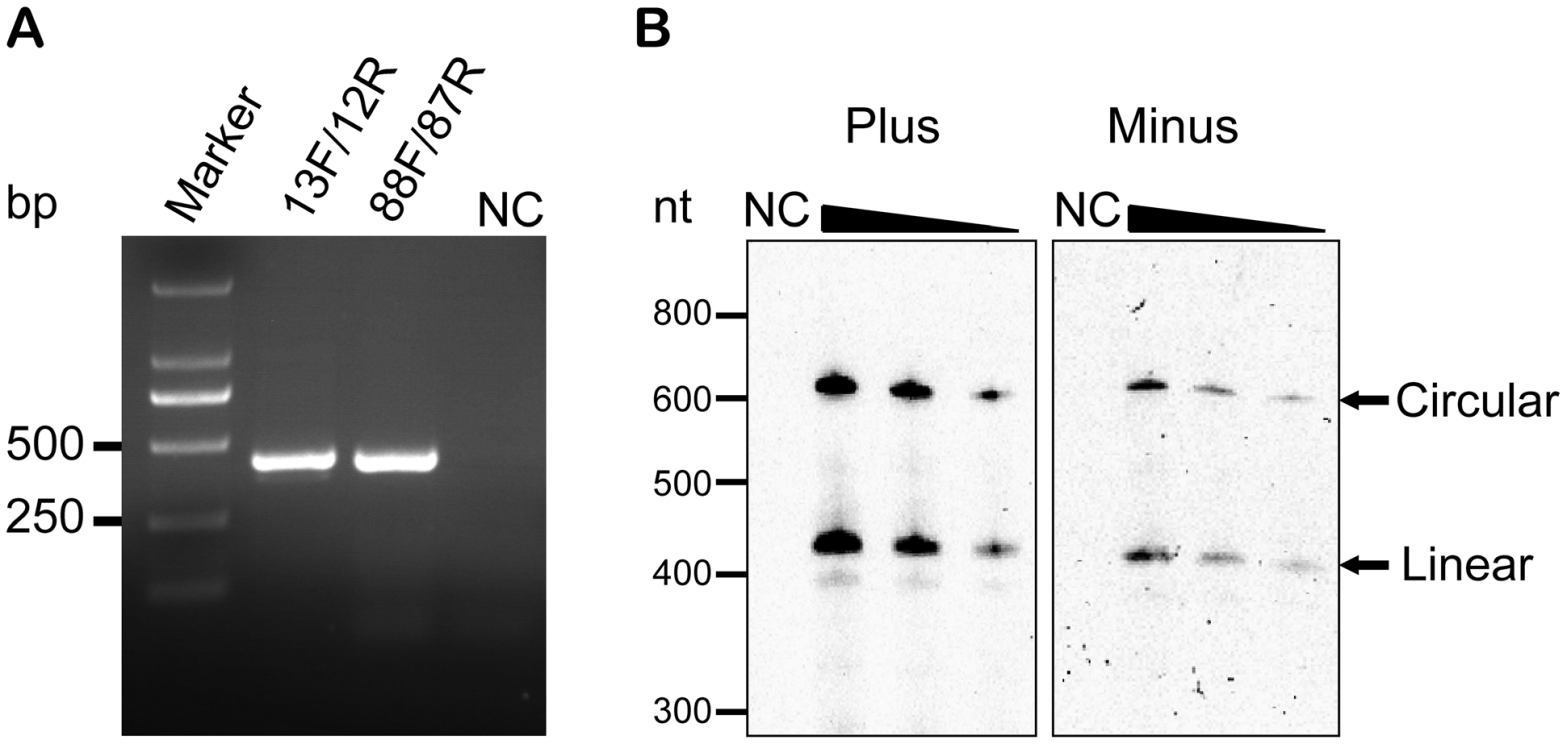
Verification of the circularity of AHVd-like RNA by RT-PCR and northern-blot hybridization. (**A**) Full length AHVd-like RNA was amplified from the diseased apple plants by RT-PCR with two pairs of primers (AHVd-88F/87R and 13F/12R), each of which is adjacent with opposite directions. ‘F’ indicates forward primers, and ‘R’ indicates reverse primers. The number before ‘F’ and ‘R’ represents locations of the 5’ end of corresponding primers in the AHVd-like RNA genome. The sample from apple used as a negative control (NC) was collected from the same orchard as the diseased apple plant. (**B**) Polarity determination of AHVd-like RNA strands by denaturing PAGE and northern-blot hybridization using two full-length ribo-probes for detecting plus (left) and minus (right) strands. The amount of both riboprobes were measured via comparison with a known concentration of DIG-labeled control nucleic acids. The amount of both riboprobes used for hybridization and the exposure time were the same. Nucleic acid preparations were diluted 10-fold. The RNA marker was indicated at the left.

To verify the predicted sequence and the circular nature of AHVd-like RNA, total RNAs from the diseased apple were isolated for divergent RT-PCR analysis. According to the sequence of AHVd-like RNA assembled by PFOR2, two pairs of adjacent primers with opposing polarities (AHVd-13F/12R and AHVd-88F/87R, sequences of primers are shown in S1 Table) were designed for RT-PCR so that the full-length AHVd-like sequences would be amplified only when AHVd-like RNA existed in a circular form. We found that RT-PCR analysis of the apple RNA sample with either primer pair yielded a single DNA species with the expected size, demonstrating the circular nature of AHVd-like RNA from the apple tree (Fig. 2A). Moreover, direct DNA sequencing of the RT-PCR products confirmed the sequence of AHVd-like RNA assembled by PFOR2.

We noted that the full-length AHVd-like RNA could be amplified when either of the primer pairs was used in RT reactions, indicating the existence of both plus and minus circular RNA molecules in the infected tissue. To further investigate the in vivo properties of AHVd-like RNAs, nucleic acid preparations were analyzed by denaturing PAGE and Northern-blot hybridization with a probe either corresponding or complementary to the assembled full-length AHVd-like RNA. The characteristic circular and linear forms were detected in the infected tissue but not in the healthy apple plant (Fig. 2B). This result further validated the in vivo circularity of AHVd RNAs. Furthermore, the AHVd-like RNAs with opposing polarities appeared to accumulate at different levels (Fig. 2B). Given that the strand accumulating at a higher level is arbitrarily assigned to be the plus polarity, the sequence of AHVd-like RNA obtained by PFOR2 was designated as the plus strand.

### Primary and proposed secondary structures of AHVd RNA

**Figure 3:**
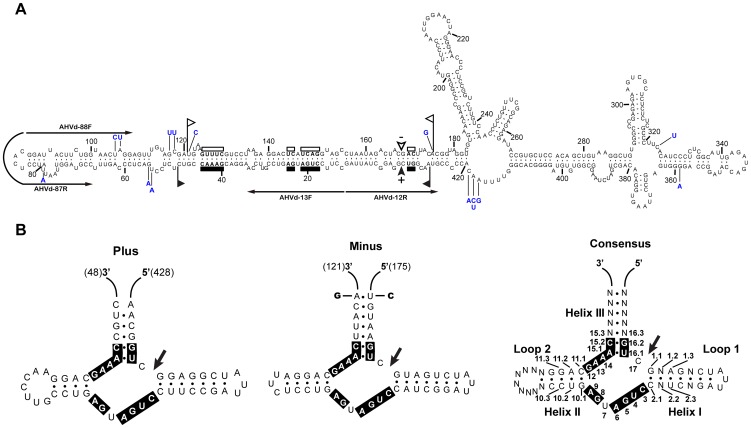
Primary and proposed secondary structures with minimum free energy for AHVd-like RNA. (**A**) The mutations observed in AHVd-like RNA variants are indicated in blue. The sequences forming the hammerhead structures are delimited by flags, the 13 nucleotides conserved in most natural hammerhead structures are denoted by bars, and the predicted self-cleavage sites are marked by arrowheads. Solid and open symbols refer to plus and minus polarities, respectively. The two pairs of arrows with opposed directions represent the primers used for amplification of the full-length AHVd-like RNA. (B) The schematic maps of plus, minus, and consensus hammerhead structures of AHVd-like RNA. The positions of nucleotides in the consensus hammerhead structure were indicated. 13 nucleotides strictly conserved in hammerhead structure are boxed and self-cleavage sites are marked by arrows. Two mutations, indicated in the minus hammerhead structure strand, do not alter the proposed secondary structure.

Cloning and sequencing of full-length cDNA clones of viroids would supply relevant information on sequence variability in the natural viroid-like RNA populations. The sequenced cDNA clones of AHVd-like RNA were amplified by the two primer pairs, AHVd-13F/12R and AHVd-88F/87R. Therefore, the putative mutations located at the positions of one pair of primers were determined through amplification and sequencing with the second pair of primers. In total, 14 sequences of AHVd-like RNA were obtained. None of these AHVd-like RNA sequences were 100% identical to other 13 sequences. However, one sequence (clone of 1–12 shown in S2 Figure) was identical to the assembled sequence of AHVd-like RNA by PFOR2. The alignment of these 14 sequences revealed the presence of 36 mutations in the population of AHVd-like RNA. Although a high-fidelity DNA polymerase was used for PCR amplification, we were not able to exclude possible errors introduced during RT-PCR. Thus, after 22 mutations detected only in one clone were removed, the remaining 14 mutations found in at least two clones were tentatively considered to be natural variations (Fig. 3A and S2 Figure). The above analyses showed that the clone of 1–12 represented consensus sequences of AHVd-like RNA, a circular molecule of 434 nt consisting of 114 G (26.3%), 116 C (26.7%), 96 A (22.1%), and 108 U (24.9%) with a G+C content of 53% (Fig. 3A).

AHVd-like RNA did not contain the characteristic central conserved region (CCR) found in the viroid family *Pospiviroidae* [15]. However, both strands of AHVd-like RNA could be folded into the conserved hammerhead ribozyme structure found in the *Avsunviroidae* and other small catalytic RNAs [25]. The predicted secondary structure of minimal free energy for AHVd-like RNA was of the quasi-rod-like class of viroids and showed two bifurcations at the right terminal part of the molecule (Fig. 3A), which was similar to that of *Eggplant latent viroid* (ELVd) [26]. The paired nucleotide residues represented 68.2% of the total, including 56.8% G:C, 35.1% A:U, and 8.1% G:U. Interestingly, 11 out of 14 observed mutations either were mapped in the loop regions or did not affect base pairing (Fig. 3A), which indirectly supported the proposed secondary structure of AHV-like RNA existing *in vivo*.

### Hammerhead structures and other features of AHVd-like RNA

Both strands of AHVd-like RNA could form natural hammerhead structures (Fig. 3B) containing 11 strictly conserved residues [27] and the adjacent helices flanking the self-cleavage sites of a group of small catalytic RNAs. In the plus and minus hammerhead structures of AHVd-like RNA, helix III was stable and helices I and II were closed by short loops 1 and 2. These features were similar to the hammerhead structures of (i) ELVd [26], (ii) PLMVd [27], (iii) satellite RNAs of the nepoviruses and sobemoviruses [9], [28], (iv) a cherry small circular RNA (csc RNA1) [29], and (v) GHVd RNA discovered very recently in grapevine [17].

The hammerhead structures of AHVd-like RNA were carefully compared with those of other known viroids and circular satRNAs (namely viroid-like RNAs), revealing some common salient features (Fig. 3B). i) In most natural hammerhead structures, positions 10.1 and 11.1 form a G-C pair, and positions 15.2 and 16.2 form a C-G pair (see ref. [30] for nomenclature). AHVd-like RNA hammerhead structures conformed to this rule. ii) A cytidylate residue preceded the predicted self-cleavage sites of AHVd RNA hammerhead structures, as occurs in most other known hammerhead structures. iii) The residue of position 7 between the conserved CUGA and GA sequences was a U in both RNA hammerhead structures of AHVd-like, which also conformed to the examples observed in most natural hammerhead structures wherein this residue is U, C, and, exceptionally, A. However, the hammerhead structures of AHVd-like RNA exhibited some peculiarities. Both hammerhead structures of AHVd-like RNA shared sequence similarities in the helices and loops with the strictly conserved helix II and loop 1 (Fig. 3B). Sequence similarities included 4 base-pairs of CAGG with CCUG, forming helix II in the consensus hammerhead structure of AHVd-like RNA (Fig. 3B), which corresponded to the equivalent positions in the consensus hammerhead structure of ELVd, the plus strand hammerhead structures of GHVd RNA [17], satellite RNAs of *Chicory yellow mottle virus* (CYMV) and *Tobacco ringspot virus* (TRSV) [31]. Moreover, loop 2 of the (+) hammerhead of AHVd-like RNA contained 7 nucleotides and was the largest reported among natural hammerheads. Importantly, the base substitutions found in different AHVd-like RNA variants in the region of the hammerhead structures did not affect the stability of–helix III and no mutations were found in helices I and II (Fig. 3B), suggesting that these self-cleaving domains were functional in vivo.

### *In vitro* self-cleavage of AHVd RNA

**Figure 4:**
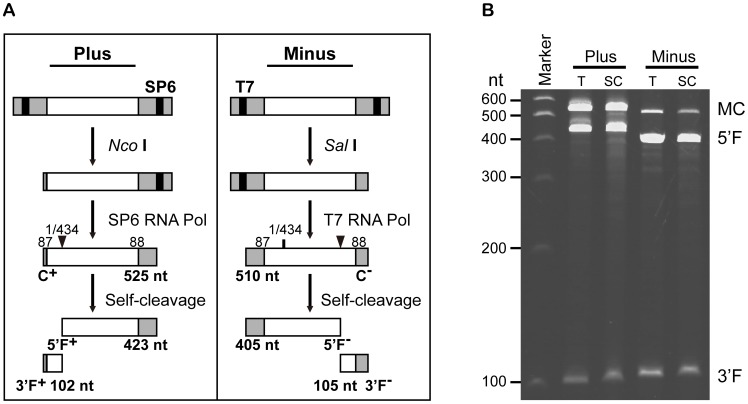
*In vitro* synthesis and self-cleavage of monomeric plus and minus RNAs of AHVd-like RNA. (**A**) Diagram of plus and minus DNA templates and of the products generated by transcription with SP6 and T7 RNA polymerases, respectively, after digestion by the indicated enzymes. Open boxes represent sequences of AHVd-like RNA, light-grey shaded boxes indicate vector sequences, and solid boxes indicate RNA polymerase promoters. The entire primary transcripts are C^+^ and C^-^, and the cleavage fragments are 5′F^+^, 3′F^+^, 5′F^-^, and 3′F^-^. Positions in the AHVd-like RNA sequence are shown above the products, and their expected sizes in nucleotides are shown below. Self-cleavage sites are indicated by arrowheads. (**B**) In vitro transcription (T) reactions and the self-cleavage (SC) reactions of purified monomeric products were separated by 5% denaturing polyacrylamide gel electrophoresis and visualized by ethidium bromide staining. MC indicates plus and minus monomers of complete transcripts of AHVd-like RNA.

The activity of the predicted ribozymes encoded by AHVd-like RNA was investigated. Full-length monomeric plus and minus AHVd-like RNA transcripts were synthesized *in vitro* from linearized plasmids and were found to be self-cleaved during transcription and after purification when incubated under standard self-cleavage conditions in a protein-free buffer (Fig. 4). The cleaved fragments (5′F and 3′F) for the plus and minus strands of the transcripts showed the expected lengths based on the predicted self-cleavage sites of the hammerhead structures (Fig. 4). The predicted cleavage sites (Fig. 3A and 3B) were also experimentally confirmed by rapid amplification of 5′-cDNA ends (5′-RACE)-PCR (S3 Figure). We noted that the plus strand full-length AHVd-like RNA transcripts were more stable during transcription than the minus strand transcripts (Fig. 4B), suggesting a higher self-cleavage efficiency of the minus strand hammerhead ribozyme.

### Detection of viruses, ASSVd and AHVd-like RNA in apple plants

Although the above analyses determined the circularity and self-cleavage activity of AHVd-like RNA, it was still unclear whether AHVd-like RNA represented a new viroid or a circular satRNA. If AHVd-like RNA corresponded to a new plant circular satRNA, it was expected that a helper virus would be present in the diseased tissues to support its replication. To this end, the sRNAs from the diseased apple tree were assembled by Velvet program [32]. BLAST analysis identified contigs that showed sequence similarities with *Apple chlorotic leaf spot virus* (ACLSV), *Apple stem grooving virus* (ASGV), and *Apple stem pitting virus* (ASPV). The presence of these three plant viruses was further confirmed by RT-PCR (S4 Figure). However, we noted that none of these three plant viruses have been reported to have satRNAs. A survey of 182 apple tree samples from different cultivars was performed to determine whether AHVd-like RNA co-existed with any of these viruses. We found that AHVd-like RNA was detected in 75 of the 182 apple tree samples. Notably, the incidence of AHVd-like RNA was not associated with ACLSV, ASPV, or ASGV, suggesting that AHVd-like RNA might be a novel viroid (S2 Table). However, the viroid nature of AHVd-like RNA remained to be verified because neither Northern-blot hybridization nor RT-PCR detected the replication of AHVd-like RNA in the apple seedlings free of AHVd-like RNA one year after mechanical inoculation with the dimeric transcripts synthesized in vitro from the full-length cDNA clones of AHVd-like RNA described above.

### Size distribution of sRNAs and mapping on the genome of AHVd-like RNA

**Figure 5:**
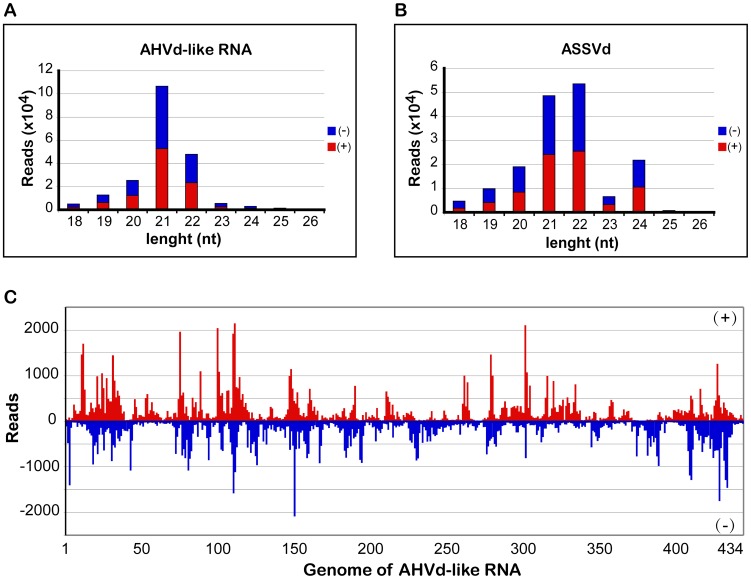
Size distribution and mapping of AHVd-like RNA-specific sRNAs on the AHVd-like RNA genome. Different sizes (18–26 nt) of sRNAs derived from AHVd-like RNA (**A**) and ASSVd (**B**) were compared with each other. The amount of plus (red) and minus (blue) sRNAs of AHVd-like RNA and ASSVd are similar. AHVd-like RNA specific reads 21 to 24 nt in length were mapped on the AHVd-like RNA genome (**C**).

Given that the size distribution of sRNAs derived from viroids might serve as an indicator of the subcellular localization or replication sites of the viroids [33], [34], we next compared the accumulation and profile of sRNAs derived from AHVd-like RNA with those of ASSVd in the same tissues of the apple tree. Similar to ASSVd, AHVd-like RNA specific sRNAs from different size families were all divided approximately equally into the plus and minus strands and the predominant sRNA species was the 21 nt class (Fig. 5A). However, few of the AHVd-like RNA-specific sRNAs were 24 nt long. A similar size distribution profile was observed for vd-sRNAs from tissues infected by PLMVd [33], [35] and GHVd [17]. In contrast, a notable amount of ASSVd sRNAs belonged to the 24 nt class (Fig. 5B), similar to several viroids that replicate in the nucleus [34], [36]–[38]. These findings suggest that AHVd-like RNA may not replicate in the nucleus. AHVd-like RNA specific sRNA reads of 21 to 24 nt in length were mapped to the corresponding positions on the AHVd-like RNA genomic or anti-genomic RNAs (Fig. 5C). As previously reported for PLMVd sRNAs isolated from infected peach [30], [32] and GHVd RNA-specific sRNAs from infected grapevine [17], the sRNAs of AHVd-like RNA were derived from every position in both the genomic and anti-genomic strands, and their distribution was biased, with a profile of several hotspots (Fig. 5C).

### Discovery of a new viroid from an old grapevine plant by PFOR2 analysis

Grapevine is a natural host for many viroids [39]. Although most of these viroids do not induce symptoms in grapevine, cultivated grapevines with latent viroid infections may serve as reservoirs for certain viroids to infect crops and cause severe diseases [40]. The discovery of a novel viroid-like circular RNA from the original ‘Pinot noir’ grapevine by PFOR [17] suggests that more novel viroids or viroid-like RNAs may exist in cultivated grapevines, especially in some old grapevines. Collections of several grapevine stocks of at least 100 years of age in Xinjiang, China [39] allowed us to test this hypothesis. Of these grapevine trees, a ‘Thompson Seedless’ plant grown in Tulufan was selected for sRNA deep sequencing and viroid discovery by PFOR2.

The obtained sRNA library contained 14,033,487 clean reads of 17–30 nt in length, with 21 nt as the most dominant size class (S5 Figure). PFOR2 analysis of the library took 2 hours and 24 minutes and was 7.1 times faster than PFOR analysis. Complete genomes of four known viroids: HpSVd, GYSVd-1, GYSVd-2, and *Australia grapevine viroid* (AGVd), which have been previously detected by RT-PCR and Northern-blot hybridization in this old grapevine tree [39], were assembled by PFOR2. PFOR2 analysis of the grapevine sRNA library also identified a putative circular RNA molecule of 328 nt in length that shared 79% sequence similarity with *Citrus viroid* VI (CVd-VI) (accession no. AB019508). Because CVd-VI had not been previously isolated from grapevine and the sequence similarity between CVd-VI and the identified RNA molecule was below the viroid species demarcation criteria of 90% sequence similarities [15], we hypothesized that the circular RNA revealed by PFOR2 represented a new viroid and was tentatively designated as Grapevine latent viroid (GLVd) hereafter.

### Circularity of GLVd

**Figure 6:**
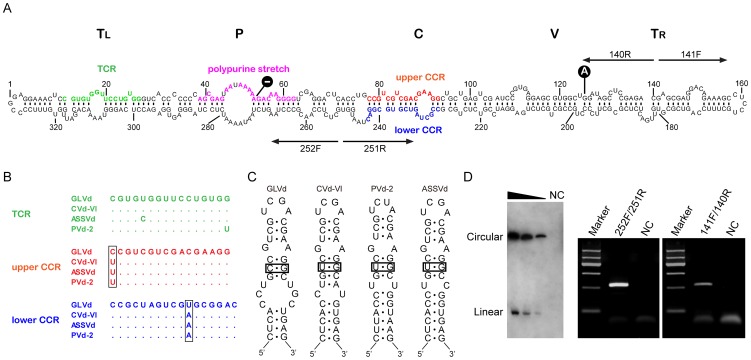
Characterization of predicted secondary structure, conserved structure elements, and circular properties of GLVd. (**A**) Variant structure domains (T_L_, P, C, V, and T_R_) are listed above the secondary structure of GLVd. The nucleotides of the upper central conserved region (CCR) and the lower CCR are indicated in red and blue, respectively. The nucleotides in green indicate the structural element of the terminal conserved region (TCR). Two mutations of GLVd are marked in dark circles. The two pairs of arrows with opposed directions represent the primers used for amplification of the full-length GLVd genome. (**B**) Exact comparisons of the conserved elements (TCR, upper CCR, and lower CCR) among GLVd, CVd-VI ASSVd, and PVd-2. The nucleotides in squares indicate the mutations of GLVd compared with the other three viroids. (**C**) The proposed structure element of the HPI in GLVd, CVd-VI, ASSVd and, PVd-2. The lower loop in the stem of the GLVd HPI is larger than those of the other three viroids. (**D**) The presence of the circular RNA of GLVd in the old grapevine sample was detected by RT-PCR with two pairs of primers, 141F/140R and 252F/251R (right). The circular and linear strands of GLVd RNAs were detected by northern-blot hybridization with full length DIG-labeled probes. A series of nucleic acids preparations were diluted 10-fold.

To confirm the viroid nature of GLVd, we first determined whether GLVd existed in a circular form in vivo. Two sets of adjacent primers of opposite polarity (GLVd-252F/251R and GLVd-141F/140R, shown in S1 Table), derived from the predicted sequence by PFOR2, were synthesized and used for the amplification of the full-length circular GLVd by RT-PCR. As a control, PCR was performed with these primers using the template of total DNA isolated from the old grapevine without the RT step to determine whether GLVd was derived from repeat elements of the host genome. Divergent RT-PCR with either of the two primer pairs yielded a product with the expected size whereas no specific products were amplified by PCR (Fig. 6D), confirming the circular RNA nature of GLVd. The amplified DNA of the expected size was eluted, and four clones from each primer pair were sequenced. Sequence analysis revealed the presence of a master sequence represented by six clones, while the two sequence variants contained a deletion of A at position 54 and a substitution (G/A) at position 125, respectively (Fig. 6A). Importantly, the master sequence of GLVd was identical to the sequence discovered by PFOR2 and was 328 nt in length, with 67 A (20.4%), 70 U (21.3%), 96 G (29.3%) and 95 C (29%), producing a G+C content of 58.3%.

The availability of the full-length GLVd genomic sequence allowed us to synthesize a GLVd-specific riboprobe for detecting various molecular forms of GLVd RNA by Northern-blot hybridization. Total RNAs extracted from the old grapevine were separated by denaturing PAGE followed by Northern-blot hybridization, leading to the detection of the characteristic circular and linear forms (Fig. 6D). These findings together indicated that GLVd existed as a circular RNA in the old grapevine tree.

### Secondary structure and conserved structure elements of GLVd

The minimal free-energy secondary structural prediction revealed a rod-like conformation of GLVd. The predicted secondary structure of GLVd was similar to that proposed for most viroids [15], [41] and contained 63.4% paired nucleotides, including 67.3%, 24.0% and 8.7% of G:C, A:U, and G:U pairs, respectively (Fig. 6A). Notably, the GLVd structure contained the central conserved region (CCR), which is the key structural element and taxonomic criterion for assigning viroids to the family *Pospiviroidae*. The sequences of upper and lower CCR of GLVd were nearly identical to that of *Apple scar skin viroid* (ASSVd) [42], the type species of the genus *Apscaviroid*. The terminal conserved region (TCR) of GLVd was also similar to that found in apscaviroids (Fig. 6A and B). Furthermore, the GLVd structure included a polypurine stretch located in the pathogenicity domain, which is conserved in the family *Pospiviroidae* [41].

Hairpin I (HPI) formed by the upper CCR strand and the flanking inverted repeat [43], [44] is a conserved structural element in the family *Pospiviroidae*. A typical HPI was identified in GLVd and included the capping palindromic tetraloop, the adjacent 3-bp stem, and the 7-bp stem interrupted by two opposite-facing nucleotides that were seemingly unpaired (Fig. 6C). However, sequence variations were noted in the HPI of GLVd compared to the known apscaviroids (Fig. 6C and S6 Figure). The nucleotide substitution of U by C at the left terminus of the upper CCR converted a G:U base-pair in the stem of HPI into a G:C base-pair, which was predicted to strengthen the stability of this structure. In contrast, a large internal loop present in GLVd HPI would weaken the stability of HPI (Fig. 6C). Detection of the conserved structural features of viroids such as CCR, TCR, and HPI in GLVd further supports the idea that GLVd is a viroid.

### Detection of GLVd infection in the grapevine

To further verify the viroid nature of GLVd, dimeric head-to-tail transcripts of GLVd were transcribed in vitro from the constructed cDNA clones of GLVd. Virus-free grapevine seedlings (cv ‘Beta’) grown in early spring were mechanically inoculated with the GLVd transcripts by slashing the stems with razor blades. Uninoculated healthy seedlings from the same batch were kept as controls. Because GLVd was undetectable by Northern-blot hybridization 3 and 6 months post inoculation, we re-inoculated the seedlings with a higher dose of GLVd transcripts and detected weak hybridization signals from 4 of the 18 inoculated grapevine plants 3 months after the secondary inoculation. To facilitate GLVd detection in the young tissues, the apical shoots of the inoculated grapevine plants were removed, and the leaves from the young lateral branches that emerged 6 months after the secondary inoculation (or 12 months after the first inoculation) were collected for RNA extraction. We found that GLVd infection became readily detectable in 6 of the 18 inoculated grapevine plants using either Northern-blot hybridization or RT-PCR. The progeny sequence was determined via DNA sequencing of the cloned RT-PCR products and was found to be the same as the inoculated GLVd transcripts. Therefore, GLVd autonomously replicated in its natural host grapevine, fulfilling the most critical criterion to be considered as a viroid.

### Phylogenetic analysis and tentative taxonomy of GLVd

**Figure 7:**
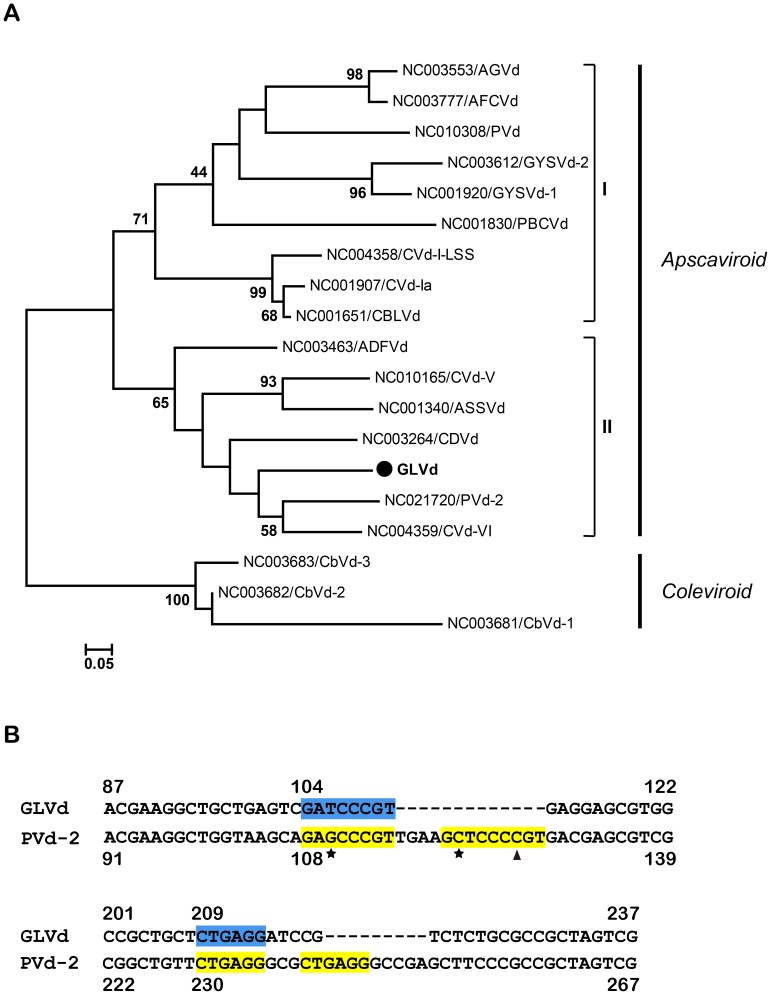
Phylogenetic analysis and alignment of GLVd. (**A**) Evolutionary relationships of GLVd with apscaviroids. The abbreviated name of viroids refer to a previous study [15]. The phylogenetic tree was constructed by MEGA 5 [73] with the neighbor-joining method. The percentage of 1000 replicate trees in which the groups clustered together in the bootstrap test is shown next to the branches. Bootstrap values less than 60% are not shown. Three viroids of Coleus viroid 1, 3, and 3 (CbVd-1, 2, and3), belonging to *Coleviroid*, were used as an out-group for phylogenetic analysis. (**B**) Alignment of partial sequences of GLVd and PVd-2 using Cluxtal W 2.0 [74] with default values. Numbers indicate the beginning and end sites of sequences above and below the sequences. The nucleotides shaded in colors represent the two simple sequence repeats (SSRs) found in GLVd and PVd-2. The nucleotides in yellow are two copies of the nucleotides in blue. Pentangles and triangles represent mutations in the SSR.

To determine the taxonomy of GLVd, the sequence of GLVd was aligned with all of the known species in the genus *Apscaviroid*. The phylogenetic tree constructed using viroids of genus *Colviroid* as the out-group revealed two subgroups of apscaviroids (Fig. 7A). GLVd was clustered in subgroup-II and most closely related to CVd-VI and a tentative new species of Persimmon viroid 2 (PVd-2) identified very recently from American persimmon (*Diospyros virginiana* L.) [45] (Fig. 7A). These results indicated that GLVd should be considered as a new member in the genus *Apscaviroid*. Interestingly, careful inspection of the apscaviroid alignments identified two types of repeat sequences between GLVd and PVd-2 (Fig. 7B). Further study is necessary to determine whether the repeat sequences were involved in host adaptation because simple sequence repeats (SSRs) distribute non-randomly in viroid genomes and might play a significant role in the evolution of viroids [46].

### Small RNA-independent discovery of biologically active circular RNAs by PFOR2 combined with a new computational program

We next developed a simple computational program, Splitting Longer reads into Shorter fragments (SLS), as part of PFOR2 to discover biologically active circular RNAs via the deep sequencing of long RNAs instead of small RNAs. The program cut the sequenced long RNAs into 21-nt virtual sRNAs of 20-nt overlap with their 5′ and 3′ neighboring sRNAs before PFOR2 analysis to retain only 21-nt virtual ISRs for the final assembly of circular RNAs (Fig. 1B). To determine the efficacy of SLS-PFOR2, we sequenced the total RNAs from PSTVd-infected potato seedlings by constructing independent libraries using Not Not So Random (NNSR) library protocol [47] after either depletion of the abundant ribosomal RNAs [48], [49] or enrichment for circular RNAs following the degradation of linear RNAs by RNase R [50] (S7 Figure). The sequencing of the rRNA-depleted library yielded 774,621 reads longer than 100 nt, among which 83 reads were derived from PSTVd with a mean length of 160 nt. A total of 92,093 reads longer than 100 nt were obtained from the RNase R-treated library, with 55 reads from PSTVd. We found that the full-length PSTVd molecule of 354 nt was readily identified by SLS-PFOR2 from both the rRNA-depleted library (k-mer 19 or 20) and the RNase R library (k-mer 17 or 18) with a running time of 3 hours 20 minutes and 103 hours 14 minutes, respectively. These results demonstrated that SLS-PFOR2 is capable of discovering circular RNAs independently of the in vivo production or the deep sequencing of their specific small RNAs.

## Discussion

Next-generation sequencing (NGS) approaches can readily identify viral and subviral pathogens in samples of plant and animal diseased tissues that are related in nucleotide sequence or encoded protein sequence to a known pathogen. The development of PFOR for viroid discovery thus represents a conceptual advance because, unlike NGS and several available classical approaches, PFOR does not depend on sequence homology with a known viroid. The major improvements described in this study overcame the limitations of the published version of PFOR that restrict its potentially widespread applications in pathogen discovery. PFOR2 was 3.3, 5.4, and 7.1 times faster than PFOR in the analysis of the three small RNA datasets from grapevine, peach, and apple, respectively. The enhanced speed is likely to be critical for viroid discovery when targeting hosts with large genome sizes and/or abundant small RNA populations. For example, our analysis of a small RNA library from Areca catechu with 46,637,488 reads took 8 hours and 40 minutes by PFOR2 instead of 110 hours by PFOR (unpublished data). The efficacy of PFOR2 was verified with the discovery of GLVd as a novel viroid that initiates independent infection in its natural host. Moreover, the development of SLS-PFOR2 eliminates the requirement for the in vivo production and accumulation of Dicer-dependent siRNAs to target the circular RNAs to be identified. As a result, small RNA sequencing becomes unnecessary, and RNA-seq libraries depleted of either ribosomal RNAs or linear RNAs can be analyzed by SLS-PFOR2 for the discovery of both replicating and non-replicating circular RNAs in diverse organisms. In principle, SLS-PFOR2 can identify novel viroid circular RNAs in host species that replicate but do not trigger the biogenesis of viroid-specific siRNAs. Therefore, SLS-PFOR2 has the potential to expand the list of both viroids and host species that support viroid infection.

PFOR and FOR2 separate small RNAs in the pool into TSR and ISR groups based on the presence of the minimal overlapping k-mer among reads and remove all TSRs progressively. As a result, the overlapping sets of small RNAs retained after the filtering process might be different when different k-mers are used, leading to the variations in the sequences assembled by PFOR and PFOR2 that may not reflect the natural heterogeneity of viroids. The successful detection of each viroid by PFOR and PFOR2 is dependent on whether the circular RNA has been completely covered by a set of overlapping small RNAs with the minimal length defined by k-mers at both ends after removing all TSRs. Because each ISR is allowed to be used only once during the assembling step, only one viroid would be revealed when two or more viroids share small RNAs with lengths defined by k-mers or longer. For example, although ASSVd was revealed by PFOR2 analysis of the apple library using k-mers in the size range of 18 to 20 nt, AHVd-like RNA was identified using k-mers of 18 or 19, but not of 20. In the grapevine sample, HpSVd was identified by PFOR2 using k-mers of 17 to 21, whereas GYSVd-1 and GYSVd-2 were each revealed with a specific k-mer most likely because the two viroids are 80% identical in sequence and share small RNAs. Therefore, it is necessary to analyze each small RNA library using PFOR2 with k-mers from 17 to 21 and to verify the assembled sequences of viroid candidates by RT-PCR and cDNA sequencing.

The evolutionary origin of viroids remains unknown [51]. However, it has been proposed that most, if not all, present viroid diseases of cultivated plants originated recently by the accidental introduction of viroids from endemically infected wild plants into susceptible cultivated plants [52]. Thus, the identification of the original wild host plants as symptomless viroid carriers may provide additional insight into possible evolutionary scenarios. Cultivated grapevines were assumed to be associated with ‘Etrog’ citron fruit, displaying citrus viroid disease symptoms as depicted in an ancient synagogue from the early 6th century C.E. in Israel [40]. This suggested that cultivated grapevines with latent infections of viroids may serve as reservoirs for viroid spreading and causing diseases in other hosts. Accordingly, the viroids that cause some epidemic diseases at present are likely to come from the originally infected grapevines. This hypothesis is consistent with the finding that the cultivated grapevines asymptomatically infected with HpSVd were considered as the origin of the hop stunt disease epidemic in commercial hops in Japan [53]. It is also possible that grapevines might harbor some unknown viroids that are yet to be identified. The discovery of a novel viroid-like circular GHVd RNA previously [17] and GLVd in this work supports this idea. GLVd is related to both CVd-VI and PVd-2, which were isolated from diseased Etrog citrons (*Citrus medica* L.) with mild petiole necrosis and leaf bending [54] and American persimmon [45], respectively. Since GLVd and PVd-2 appear to originate from a common ancestor, it will be of interest to determine in future studies if the two repeated sequences detected between GLVd and PVd-2 (Fig. 7B) play a role in host adaptation during transmission from its original host to certain new susceptible hosts.

Our conclusion that GLVd is a novel viroid is supported by the molecular and biological evidence presented here including its circularity, typical structural elements of viroids, and self-replication in grapevine seedlings. The phylogenetic analysis indicates that GLVd is a member of the genus *Apscaviroid*. Although we found that GLVd was able to independently infect grapevine seedlings (cv ‘Beta’), the accumulation of GLVd was low, and no obvious symptoms were observed in infected grapevine plants. Although future studies on biological properties of GLVd may further differentiate this viroid from those previously reported, the conserved structural elements, the low sequence identity (maximum of 79% with CVd-IV) with other members in the genus *Apscaviroid*, and the natural host of GLVd, strongly support the possibility of annotating it as a new species in the genus *Apscaviroid*.

It is currently unclear whether AHVd-like RNA is a viroid or a satellite RNA, in contrast to GLVd. AHVd-like RNA shared no homology with the apple genome and was not amplified by PCR without a RT step, indicating that AHVd-like RNA was exogenous. Given that AHVd-like RNA encoded self-cleavage activities and was not specifically associated with any of the viruses identified in apple trees, we propose that AHVd-like RNA is a viroid. However, we were unable to demonstrate independent infectivity in apple seedlings for either the in vitro transcripts from dimeric AHVd-like RNA cDNA clones or AHVd-like RNA purified from apple tissues. In this regard, AHVd-like RNA may be related to ASSARNA-2, a circular RNA that was previously isolated from diseased apple plants in Japan and China, known to migrate more slowly than the 330-nt ASSVd RNA and unable to establish independent infection in apple seedlings [55]–[57]. Furthermore, our search for the conserved tertiary structure of a kissing loop, which is found in most *Avsunviroidae* viroids [58], [59] and in GHVd RNA [17], identified only weak kissing loops of 3 base-pairs in AHVd-like RNA (S8 Figure). Therefore, we cannot rule out the possibility that AHVd-like RNA is a novel satRNA. However, we note that virus-derived siRNAs produced by the antiviral Dicer of a fungal host are predominantly within the 20- to 22-nt range with a peak at 21-nt [60]. It is therefore less likely that AHV-like RNA replicates and triggers Dicer recognition in a fungal host since 21- and 22-nt small RNAs derived from AHVd-like RNA were clearly more abundant than 20-nt and the remaining size classes of small RNAs as found for plant viral siRNAs produced hierarchically by Dicer-like 4 (DCL4) and DCL2 [61].

## Materials and Methods

### Plant materials

For the initial identification of viroids and viroid-like RNAs from apple, leaves were collected from an apple (*Malus pumila* Mill. cv. Fuji) plant, the fruits of which showed typical symptoms of apple scar skin viroid disease, in Shandong province China, in July 2012. The grapevine (*Vitis vinifera* L.) leaf samples used for determination of GLVd were from Tulufan in Xinjiang China. This grapevine plant (cv. Thompson seedless) for sample collections is more than 100 years old. Young leaves of both apple and grapevine were immediately put into RNAlater stabilization solution (Ambion, USA) after collection and sent to a laboratory for deep sequencing analysis. Moreover, approximately 10 g of apple and grapevine leaves were packaged with ice, kept fresh at low temperature, and sent to a laboratory for RNA analysis using PAGE and northern-blot hybridization.

To survey the occurrence of viroid-like apple RNA in China, 182 leaf samples of variant apple cultivars from five provinces were collected from 2012 to 2014 and kept at −80°C.

### Extraction and preparation of nucleic acids

Total RNAs used for deep sequencing analysis were extracted by TRIzol reagent (Invitrogen) following the manufacturer's instructions. The integrity of the resulting RNA preparations was evaluated before preparing cDNA libraries using an Agilent Technologies 2100 bioanalyzer. For RNA analysis by PAGE and northern-blot hybridization, nucleic acid preparations were obtained with buffer-saturated phenol followed by ethanol precipitation, as reported previously [62]. Methoxyethanol and CTAB were used to remove polysaccharides during purification [62].

To prepare templates of RT-PCR amplification performed for cloning full-length sequence of viroid-like apple RNA, the obtained crude extracts were run on a non-denaturing 5% polyacrylamide gel stained with ethidium bromide, and the region of the gel delimited by the 250-bp and 400-bp DNA markers was excised and eluted as described previously [26].

In the experiment involving the RNA-seq of the potato samples, the extracted total RNAs by TRIzol reagent were purified by depleting ribosomal RNAs and non-circular RNAs. RNA species smaller than 200 nt, such as 5S ribosomal RNA, were first removed using the RNeasy Mini Kit (Qiagen, USA), and 28S and 18S ribosomal RNAs were depleted by hybridization with specific probes following the instructions for the RiboMinus Plant Kit (Invitrogen, USA). To enrich circular RNAs, the total RNAs from the same sample were digested with RNase R (Epicentre, USA) at 37°C for 90 min to remove non-circular RNAs.

### RNA analysis by PAGE and northern-blot hybridization

The RNA extracts were separated using two-dimensional PAGE (2D-PAGE) under non-denaturing and denaturing conditions and stained with ethidium bromide, as previously described [63]. To determine the circularity of RNAs, the total RNAs were run on denatured PAGE gel containing 8 M urea and then transferred to Hybond N+ nylon membranes by upward capillary transfer in 20×SSC buffer. The hybridization was performed at 68°C overnight by specific probes that were generated by a DIG RNA labeling kit (Roche) according to the manufacturer's instructions. The immunological detection was performed by adding chemiluminescent substrate to the membrane following the manufacturer's instructions.

### Deep sequencing and bioinformatics analyses

The small RNA libraries were constructed using Illumina's small RNA sample preparation Kit (Invitrogen, USA) following Illumina's method. The protocols for sRNA purification, adaptor ligation, RT-PCR amplification, library purification and high-throughput DNA sequencing on an Illumina HiSeq-2000 have been reported previously [64], [65]. Two sRNA libraries of an old grapevine plant and an apple tree were sequenced. Raw data from the Illumina platform were first processed to trim adaptor and barcode sequences. Reads of 18-30 nt were extracted from the obtained trimmed reads to generate sRNA libraries for assembly. The sRNA library of a peach tree infected with PLMVd (accession no. GSM465746) and the small RNA library from a grapevine tree cultivar Pinot noir ENTAV115 (accession no. GSE18405) were downloaded from the NCBI Gene Expression Omnibus (GEO) database. All of the prepared sRNA libraries were fed into an in-house pipeline. Briefly, exogenous sRNA was enriched by subtracting sRNA derived from the host genome using the Bowtie2 with default parameters [66]. The highly enriched exogenous siRNA from each sample were assembled de novo using Velvet [32] and PFOR [17]/PFOR2. The resulting contigs were queried against the GenBank nt and nr databases using the BLAST program [67].

The RNA-seq libraries of potato samples were constructed with a modified Not Not So Random (NNSR) sequencing method [47]. Two libraries of potato were sequenced using an Ion-torrent sequencer according to the manufacturer's instructions.

### Development of the PFOR2 and SLS programs

PFOR in the PERL language was converted to PFOR2 initially by using the C++ language. OpenMP is an Application Programming Interface (API) that supports multi-platform shared memory multiprocessing programming [24]. The parallel programming technology OpenMP was employed by PFOR2 to parallelize the filtering process of singletons and TSRs concurrently. Vector was also used in PFOR2 to store all sequences temporarily in the filtering process to simplify OPENMP parallelization. In PFOR, a two-level hash table was built at each iteration process to store all sequences in the pool, whereas in PFOR2, a two-level hash table was only established at the first iteration, and non-ISRs were deleted from the two-level hash table for each subsequent iteration.

The SLS (Splitting Longer read into Shorter fragments) program was developed to cut longer reads into virtual sRNAs. The final number of generated virtual sRNAs was dependent on two metrics: sRNA size and overlap size between neighboring sRNAs (step size). Typically, a longer read was cut into contiguous 21-nt sRNAs covering the whole read, in which each sRNA overlapped 20 nt with its 5′ and 3′ neighboring sRNAs (step size  =  1).

### RT-PCR, cloning, and sequencing

The primers of HpSVd, GYSVd-1, GYSVd-2 and AGVd were described previously [39]. The primer sets for amplification of GLVd and viroid-like apple RNA were designed from the corresponding sequences of contigs assembled by PFOR2 and were listed in Supplemental Table 1. The first-strands of cDNAs were synthesized with Mu-MLV reverse transcriptase (Promega) at 42°C for 1 h, and PCR amplification was performed by high-fidelity *pfu* DNA polymerase (Thermo, USA) to generate full-length sequences of viroids and viroid-like RNA. The products of RT-PCR amplification were ligated with additional adenine (A) at the end using *Taq* DNA polymerase (Takara, Dalian) and cloned into pGEM-T vectors (Promega) with protruding 3′-terminal thymine (T). The recombinant plasmids were amplified by transforming DH5α *Escherichia coli* cells, and positive clones were randomly selected for sequencing.

### *In vitro* transcription, ribozyme cleavage analysis, and 5'RACE

The sequenced recombinant plasmids containing full-length of AHVd-like cDNA amplified with primers of AHVd-88F and AHVd-87R were digested with *Nco* I or *Sal* I to generate linear plasmids. RNA transcripts in both orientations were synthesized by T7 and SP6 RNA polymerase as described previously [29], [68]. The products of *in-vitro* transcription were purified by RNeasy Mini Kit (Qiagen, USA). The purified transcripts were incubated at 37°C for 1 h and then separated by 5% denaturing PAGE containing 8 M urea and visualized by ethidium bromide staining. Full-length of AHVd transcripts and the longer fragments resulting from their in vitro self-cleavage were excised from the gels and eluted, separately. The ribozyme activities of the purified transcripts were assessed according to previously described methods [29], [68]. The purified longer fragments were used to validate the self-cleavage sites of AHVd-like RNA by 5′RACE amplification, which was conducted using the 5′RACE System for Rapid Amplification of cDNA Ends kit (Invitrogen).

### Infectious clones of GLVd and AHVd and their bioassays

Head-to-tail dimmers of the entire sequence of GLVd and AHVd-like RNA were prepared by ligation of unit-length inserts and cloning into pGEM-T vectors (Promega), as described previously [69]. The orientation of the inserts of dimeric cDNAs was validated by sequencing. The resulting recombinant plasmids were digested into linear forms and used to synthesize dimeric transcripts with positive polarity by T7 RNA polymerase (Promega). The dimeric transcripts of GLVd and AHVd-like RNA were mechanically inoculated into grapevine (cv ‘Beta’) and apple virus-free seedlings (cv ‘Fuji’), respectively, by slashing the stems with razor blades. Each seedling was inoculated with at least 500 ng of dimeric transcripts. The inoculated seedlings were grown in a common greenhouse. The infectivity of the infectious clones of GLVd and AHVd-like RNA was examined by northern-blot hybridization every three months.

### Prediction and analysis of secondary structures

The secondary structures with minimum free energy for GLVd and AHVd-like RNA were predicted by the circular version of the MFold program [70]. The obtained secondary structures were further edited for print by RnaViz 2 [71]. To search for possible kissing-loops in AHVd-like RNA, the Kinefold web server [72] was used with default parameters.

## Supporting Information

S1 Figure **Size distribution of sRNA library from the diseased apple plant.** The total reads and the non-redundant reads are summarized separately. For total reads, reads of 21 and 22 nt are most in the library; however, among the unique reads, reads of 24 nt are predominant.
(TIF)

S2 Figure **Multiple alignments of 14 variants of AHVd-like RNA.** The full length sequences of AHVd-like RNA were amplified by RT-PCR with the primers AHVd-13F and AHVd-12R. The sequences forming the hammerhead structures are delimited by flags and the predicted self-cleavage sites are marked by arrowheads. Solid and open symbols refer to plus and minus polarities, respectively.
(TIF)

S3 Figure **Confirmation of the predicted self-cleavage sites of AHVd-like RNA by 5' RACE-PCR and sequencing.** The 3′F^+^ and 3′F^-^ fragments of AHVd-like RNA were eluted and purified from 5% denaturing polyacrylamide gel shown in Fig. 4B and were amplified by 5′ RACE-PCR. The DNA products were ligated with pGEM-T vectors and the positive clones were sequenced by Sanger sequencing. Nucleotides in yellow background represent the primer used for 5′ RACE-PCR and nucleotides in blue background represent pGEM-T vector. Cleavage sites of both polarities of AHVd-like RNA were marked with arrows.
(TIF)

S4 Figure **RT-PCR detections of ACLSV, ASPV, and ASGV.** Lane 1, the negative control; lane 2, the diseased apple plant for deep sequencing; lane 3, the positive control. The primers used in RT-PCR reactions are shown in S1 Table.
(TIF)

S5 Figure **Size distribution of the sRNA library from the old grapevine sample.**
(TIF)

S6 Figure **HPI structures of some apscaviroids.**
(TIF)

S7 Figure **Enrichment of circular RNAs from total RNA and the flow chart of the NNSR method.** (A) Depletion of 28S and 18S rRNAs by hybridization using specific probes. Equal amounts of total RNA before and after rRNA hybridization were visualized by ethidium bromide staining. (B) Digestion of linear RNAs by RNase R. Equal amounts of total RNA before and after RNase R digestion were checked on agarose gels. (C) Flow chart of the NNSR method. The RNA samples after RNase R digestion or rRNA depletion were used for first-strand synthesis primed by an adaptor A-tagged random hexamer primer (shown in blue). After RNase H treatment, the second-strand cDNA was synthesized using an adaptor B-tagged random hexamer primer (shown in yellow). Then, the library was amplified with adaptor A and adaptor B, the approximate size of amplicons were selected, and the amplicons were purified for sequencing.
(TIF)

S8 Figure **Proposed kissing loops in AHVd-like RNA.** The predicted interactions between loops are indicated with red lines.
(TIF)

S1 Table **Sequences of the primers used in this study.**
(XLS)

S2 Table **Survey of the occurrence of ACLSV, ASGV, ASPV, and AHVd-like RNA in the field by RT-PCR.**
(DOC)
