# Supplementary figures and images for: Discovery of Replicating Circular RNAs by RNA-Seq and Computational Algorithms
Source: PLoS Pathog. 2014 Dec 11;10(12):e1004553. doi: 10.1371/journal.ppat.1004553 (PMC4263765; doi:10.1371/journal.ppat.1004553)

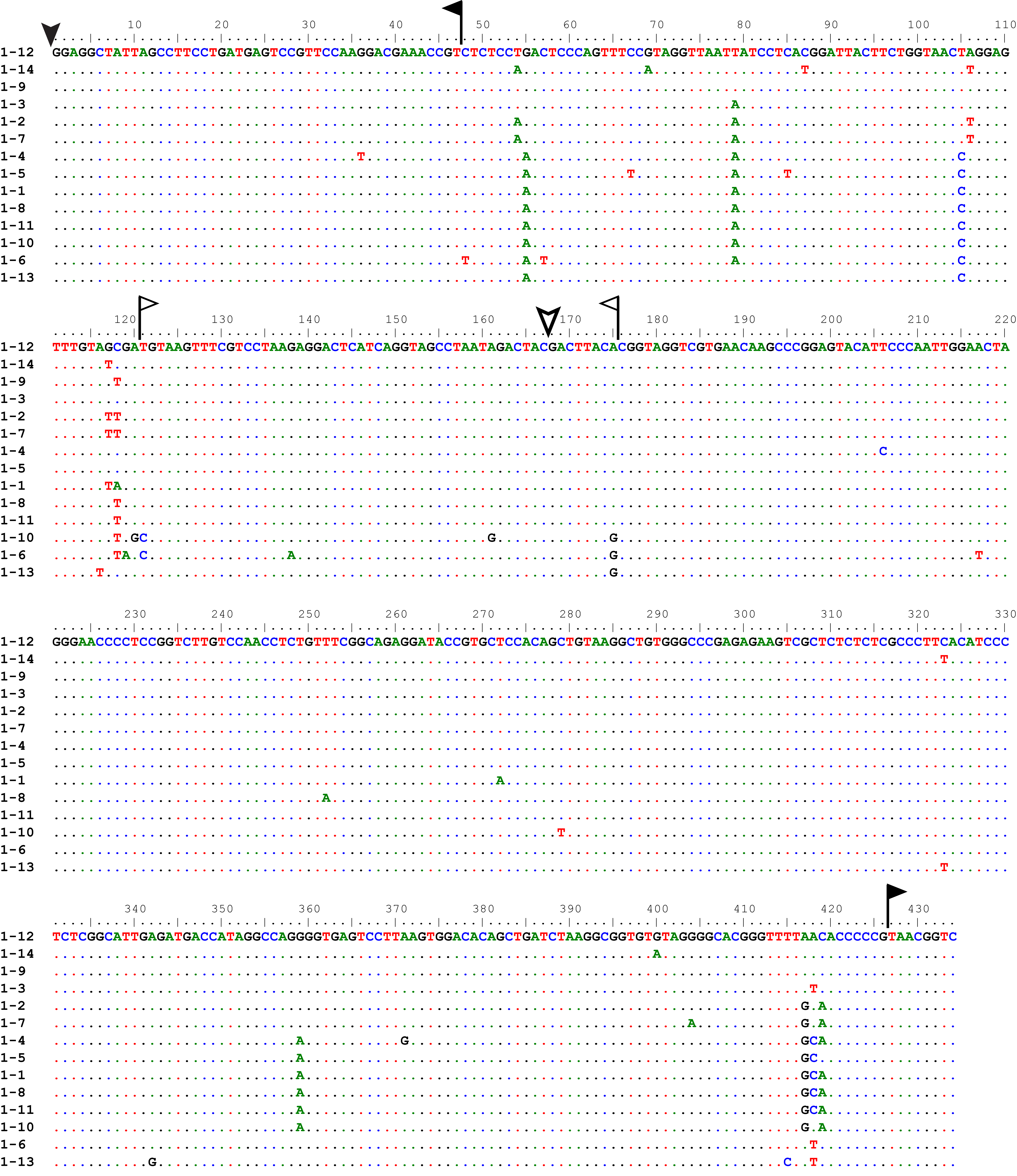

Supplement: S2 Figure — Multiple alignments of 14 variants of AHVd-like RNA. The full length sequences of AHVd-like RNA were amplified by RT-PCR with the primers AHVd-13F and AHVd-12R. The sequences forming the hammerhead structures are delimited by flags and the predicted self-cleavage sites are marked by arrowheads. Solid and open symbols refer to plus and minus polarities, respectively. (TIF) [file ppat.1004553.s002.tif]

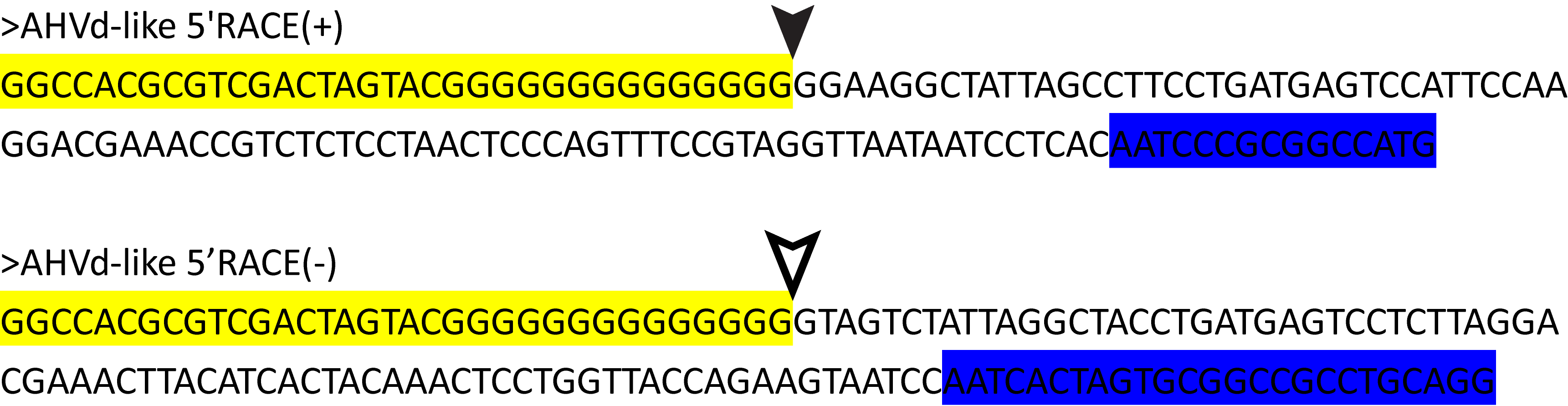

Supplement: S3 Figure — Confirmation of the predicted self-cleavage sites of AHVd-like RNA by 5' RACE-PCR and sequencing. The 3′F+ and 3′F- fragments of AHVd-like RNA were eluted and purified from 5% denaturing polyacrylamide gel shown in Fig. 4B and were amplified by 5′ RACE-PCR. The DNA products were ligated with pGEM-T vectors and the positive clones were sequenced by Sanger sequencing. Nucleotides in yellow background represent the primer used for 5′ RACE-PCR and nucleotides in blue background represent pGEM-T vector. Cleavage sites of both polarities of AHVd-like RNA were marked with arrows. (TIF) [file ppat.1004553.s003.tif]

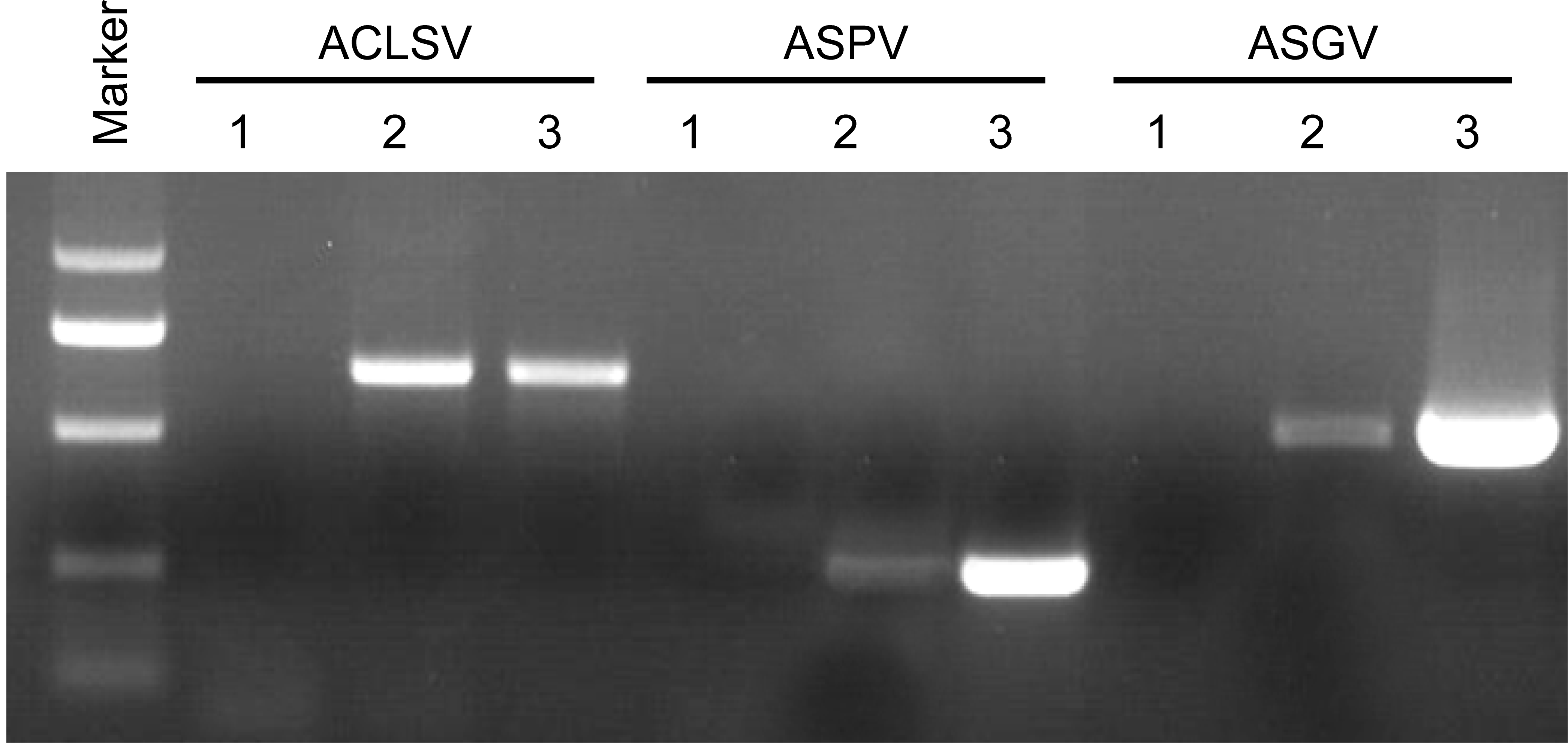

Supplement: S4 Figure — RT-PCR detections of ACLSV, ASPV, and ASGV. Lane 1, the negative control; lane 2, the diseased apple plant for deep sequencing; lane 3, the positive control. The primers used in RT-PCR reactions are shown in S1 Table. (TIF) [file ppat.1004553.s004.tif]

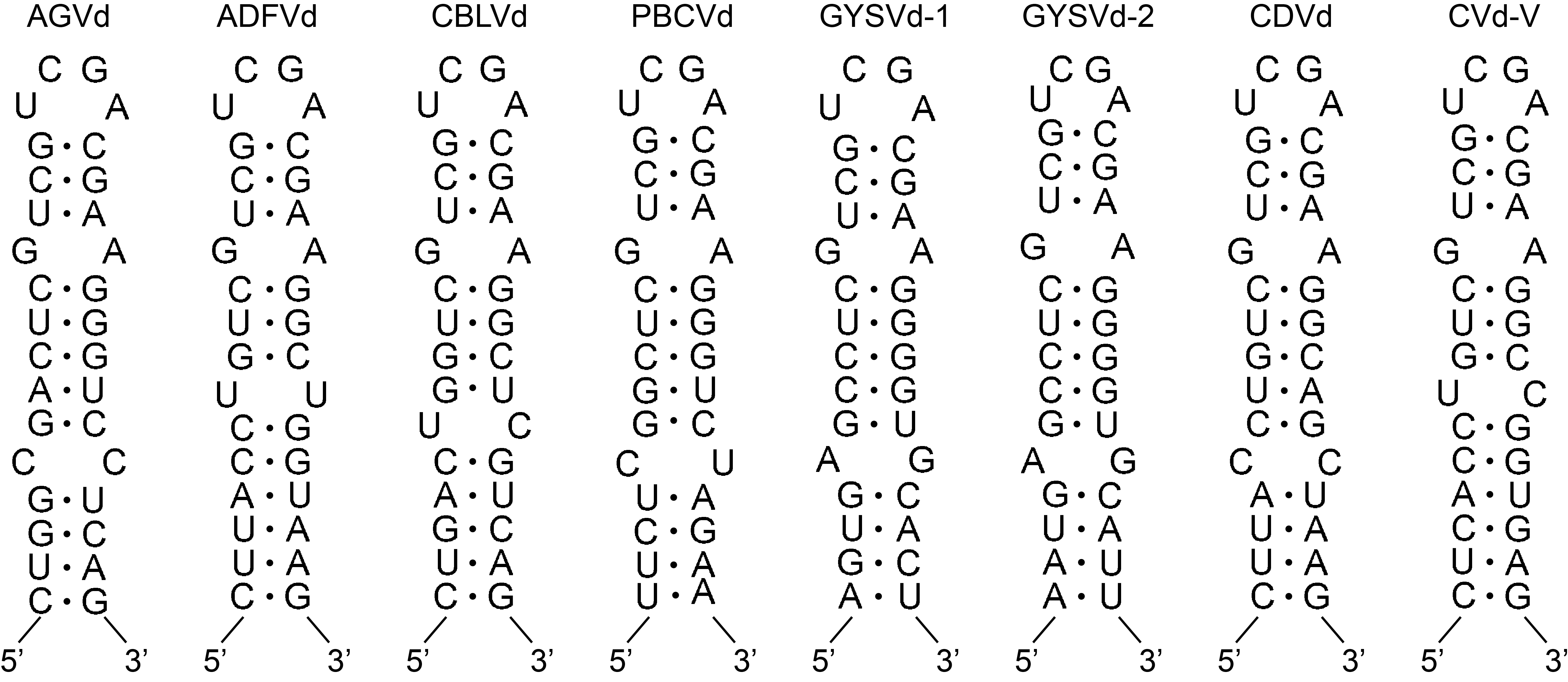

Supplement: S6 Figure — HPI structures of some apscaviroids. (TIF) [file ppat.1004553.s006.tif]

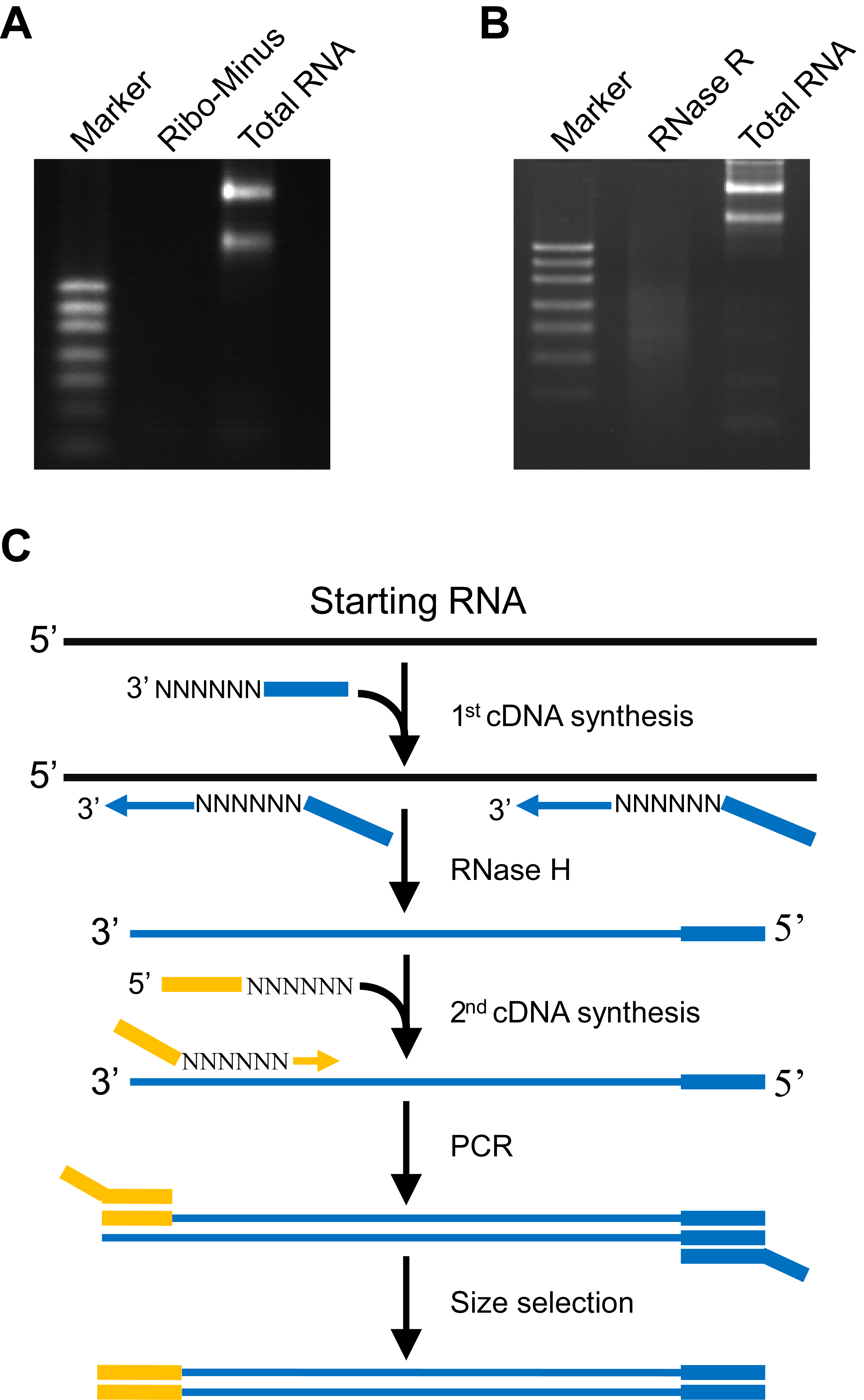

Supplement: S7 Figure — Enrichment of circular RNAs from total RNA and the flow chart of the NNSR method. (A) Depletion of 28S and 18S rRNAs by hybridization using specific probes. Equal amounts of total RNA before and after rRNA hybridization were visualized by ethidium bromide staining. (B) Digestion of linear RNAs by RNase R. Equal amounts of total RNA before and after RNase R digestion were checked on agarose gels. (C) Flow chart of the NNSR method. The RNA samples after RNase R digestion or rRNA depletion were used for first-strand synthesis primed by an adaptor A-tagged random hexamer primer (shown in blue). After RNase H treatment, the second-strand cDNA was synthesized using an adaptor B-tagged random hexamer primer (shown in yellow). Then, the library was amplified with adaptor A and adaptor B, the approximate size of amplicons were selected, and the amplicons were purified for sequencing. (TIF) [file ppat.1004553.s007.tif]

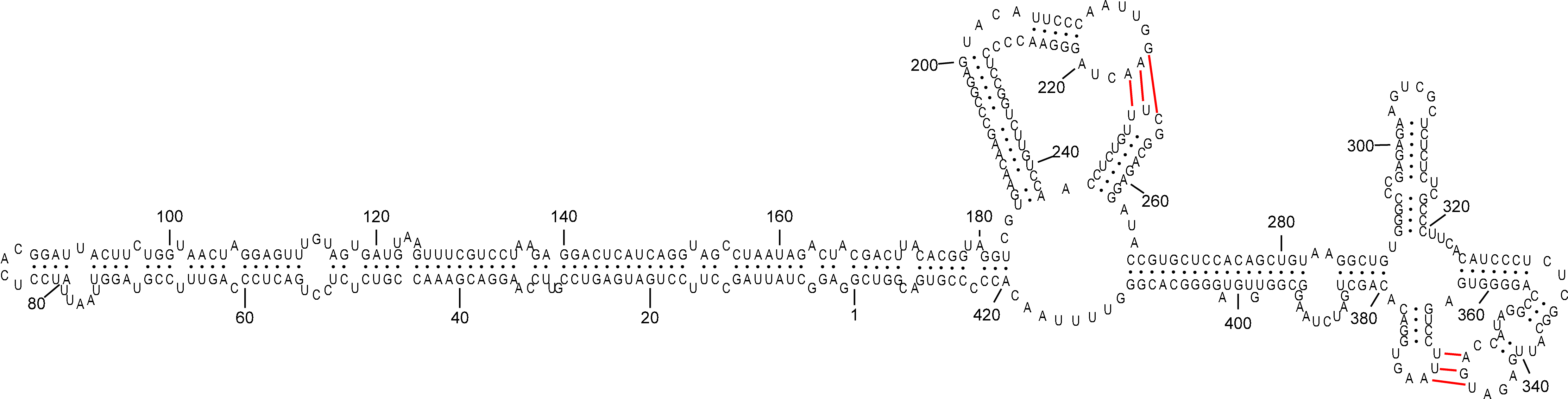

Supplement: S8 Figure — Proposed kissing loops in AHVd-like RNA. The predicted interactions between loops are indicated with red lines. (TIF) [file ppat.1004553.s008.tif]
