# Supplementary material for: Discovery of Replicating Circular RNAs by RNA-Seq and Computational Algorithms
Source: PLoS Pathog. 2014 Dec 11;10(12):e1004553. doi: 10.1371/journal.ppat.1004553 (PMC4263765; doi:10.1371/journal.ppat.1004553)
Supplement: S2 Table — Survey of the occurrence of ACLSV, ASGV, ASPV, and AHVd-like RNA in the field by RT-PCR. (DOC) [file ppat.1004553.s010.doc]

Table S2. Survey of the occurrence of ACLSV, ASGV, ASPV, and AHVd-like RNA in the field by RT-PCR.

| **Sample collection** | **Total No.** | **No. of positive samples** | | | |
| --- | --- | --- | --- | --- | --- |
|  |  | **ACLSV** | **ASGV** | **ASPV** | **AHVd-like RNA** |
| Shandong | 78 | 27 | 31 | 11 | 35 |
| Hebei | 6 | 0 | 3 | 0 | 5 |
| Liaoning | 54 | 21 | 19 | 7 | 10 |
| Beijing | 39 | 5 | 25 | 14 | 21 |
| Shaanxi | 5 | 1 | 0 | 1 | 4 |
| Total | 182 | 55(30.2%) | 78(42.9%) | 33(18.1%) | 75(41.2%) |
